# Supplementary material for: Determinants of Adherence in Time-Restricted Feeding in Older Adults: Lessons from a Pilot Study
Source: Nutrients. 2020 Mar 24;12(3):874. doi: 10.3390/nu12030874 (PMC7146127; doi:10.3390/nu12030874)
Supplement: Supplementary file 1 [file nutrients-12-00874-s001.zip › Supplementary Files/Figure S1 Time to Eat pilot study Weekly follow-up.pdf]

Study # IRB201801293 Participant ID: ______________

Caller: [| |] Date: ___/___/_______ Intervention week ___

Time to Eat pilot study – Weekly follow-up progress notes

Was the participant available? _____________________________________

Did the participant complete the entire interview? _____________________________________

Interview Questions

1. Have there been any changes in your health or lifestyle since the last time we have seen you (if first follow up call) / spoke?

____

____

2. How are you feeling *today*?

____

____

2a. How have you been feeling this *past week*?

____

____

3. Have you been completing the food diary we provided to you every day? YES / NO
What was the first time you had food or drinks with calories? What was the last time you had food or drinks with calories?

**Week ___**	**Time of 1st caloric intake**	**Time of last caloric intake**
Day 1		
Day 2		
Day 3		
Day 4		
Day 5		
Day 6		
Day 7		

* Note day of week

IF NO. Can you tell me what prevented you from completing the form?

____

4. Remind participant to follow the fasting chart provided at the first visit for allowed/restricted foods to ensure they are truly fasting.

____

____

5. Have you encountered any problems following this new eating pattern? If YES, help the participant identify specific challenges and potential solutions.

____

____

6. Do you have any questions for me at this time? Remind participant they can contact us if needed.

____

____

Additional comments:

____

____

____

Next call or final visit scheduled for: ______________________________
