# Supplementary material for: Determinants of Adherence in Time-Restricted Feeding in Older Adults: Lessons from a Pilot Study
Source: Nutrients. 2020 Mar 24;12(3):874. doi: 10.3390/nu12030874 (PMC7146127; doi:10.3390/nu12030874)
Supplement: Supplementary file 1 [file nutrients-12-00874-s001.zip › Supplementary Files/Figure S2 Exit Interview Questions.pdf]

Study # IRB201801293 Participant ID: _______________

Coordinator: ☐☐☐ Date: ___/___/_______

Exit Interview

1. What was most helpful in getting you through the fasting periods?

____
____
____
____
____

2. What, if any, difficulty did you have adhering to intermittent fasting?

____
____
____
____
____

3. Could you think of any changes to the intermittent fasting eating pattern that would make it more feasible for people to practice?

____
____
____
____
____

4. Was there any aspect of the study that you liked?

____
____
____
____
____

5. Was there any aspect of the study that you did not like?

____

____

____

____

____

6. Do you think the amount of food you ate during the study was comparable to your normal intake?
   Yes / No *Comments:*

____

____

____

____

7. Would you be willing to participate in a future study in which you intermittently fast for longer than a month involving the same eating and fasting time periods each day?

   Yes / No *Comments:*

____

____

____

____

8. Would you be willing to participate in a similar future study which included intermittent fasting and a program to increase your level of activity throughout the day?

   Yes / No *Comments:*

____

____

____

____
