# Supplementary figures and images for: Determinants of Adherence in Time-Restricted Feeding in Older Adults: Lessons from a Pilot Study
Source: Nutrients. 2020 Mar 24;12(3):874. doi: 10.3390/nu12030874 (PMC7146127; doi:10.3390/nu12030874)

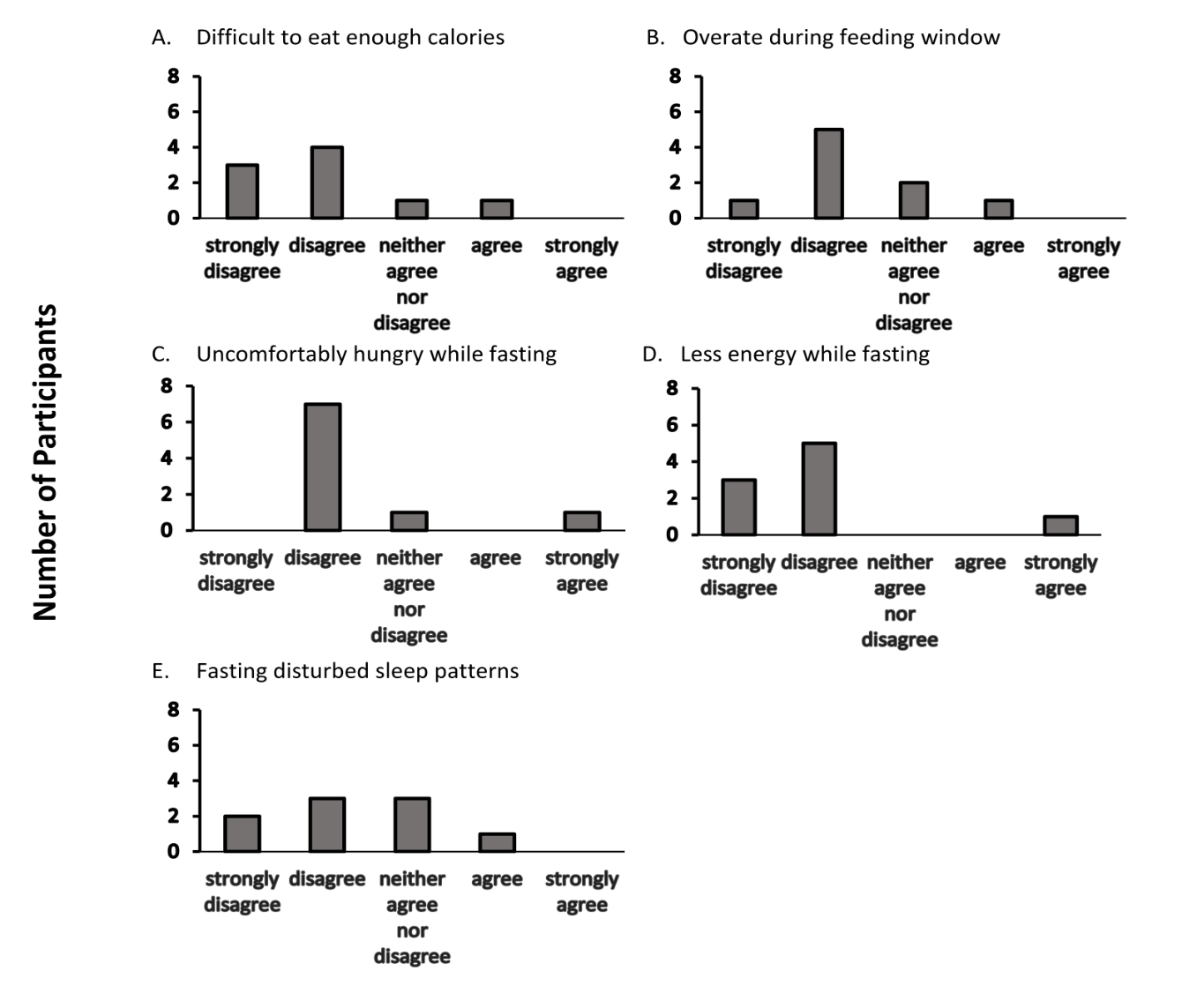

Supplement: Supplementary file 1 [file nutrients-12-00874-s001.zip › Supplementary Files/Figure S3 Participant responses to the five questions within the biological domain of the Diet Satisfaction Survey.png]

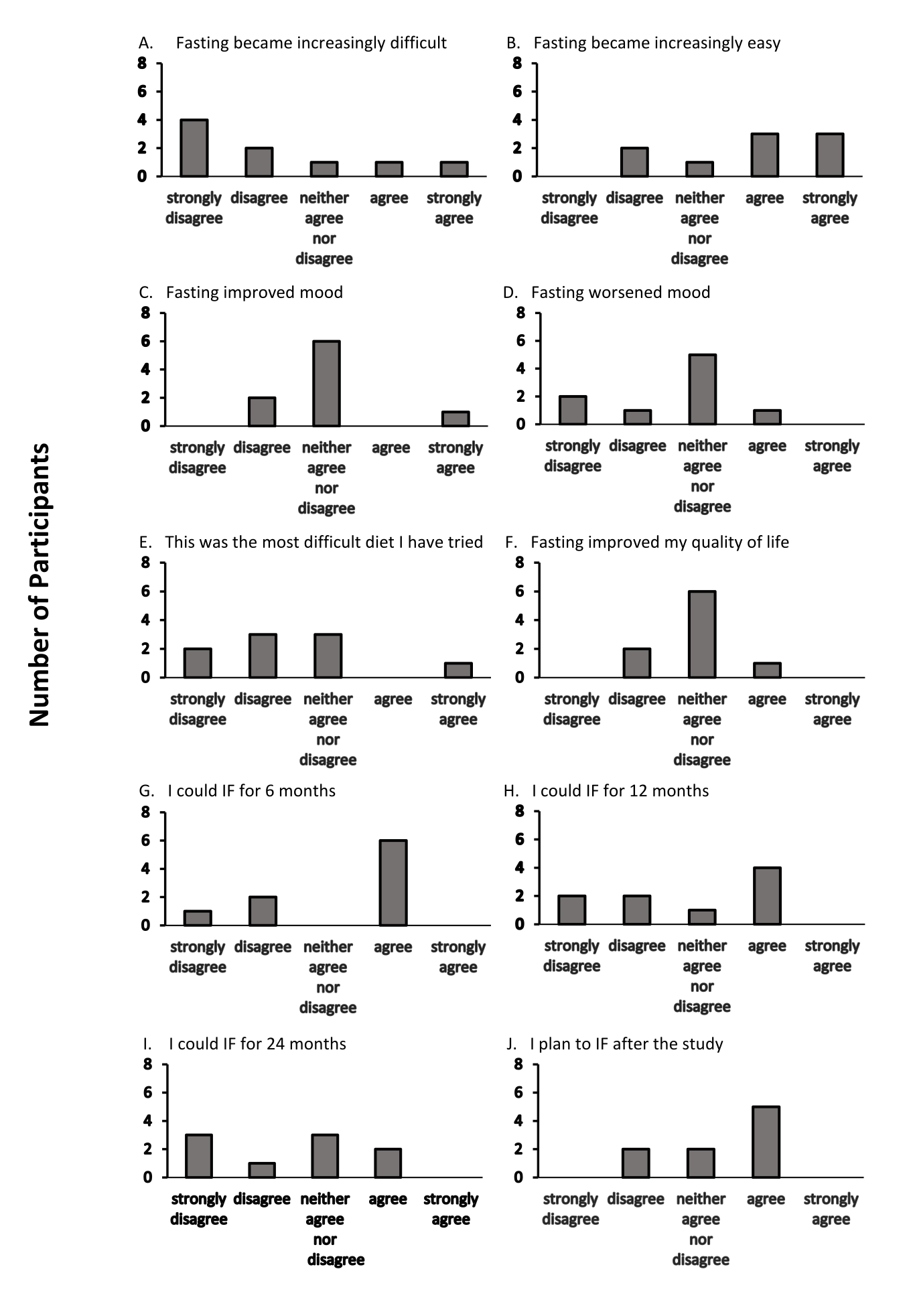

Supplement: Supplementary file 1 [file nutrients-12-00874-s001.zip › Supplementary Files/Figure S4 Participant responses to the ten questions within the psychological domain of the Diet Satisfaction Survey.png]

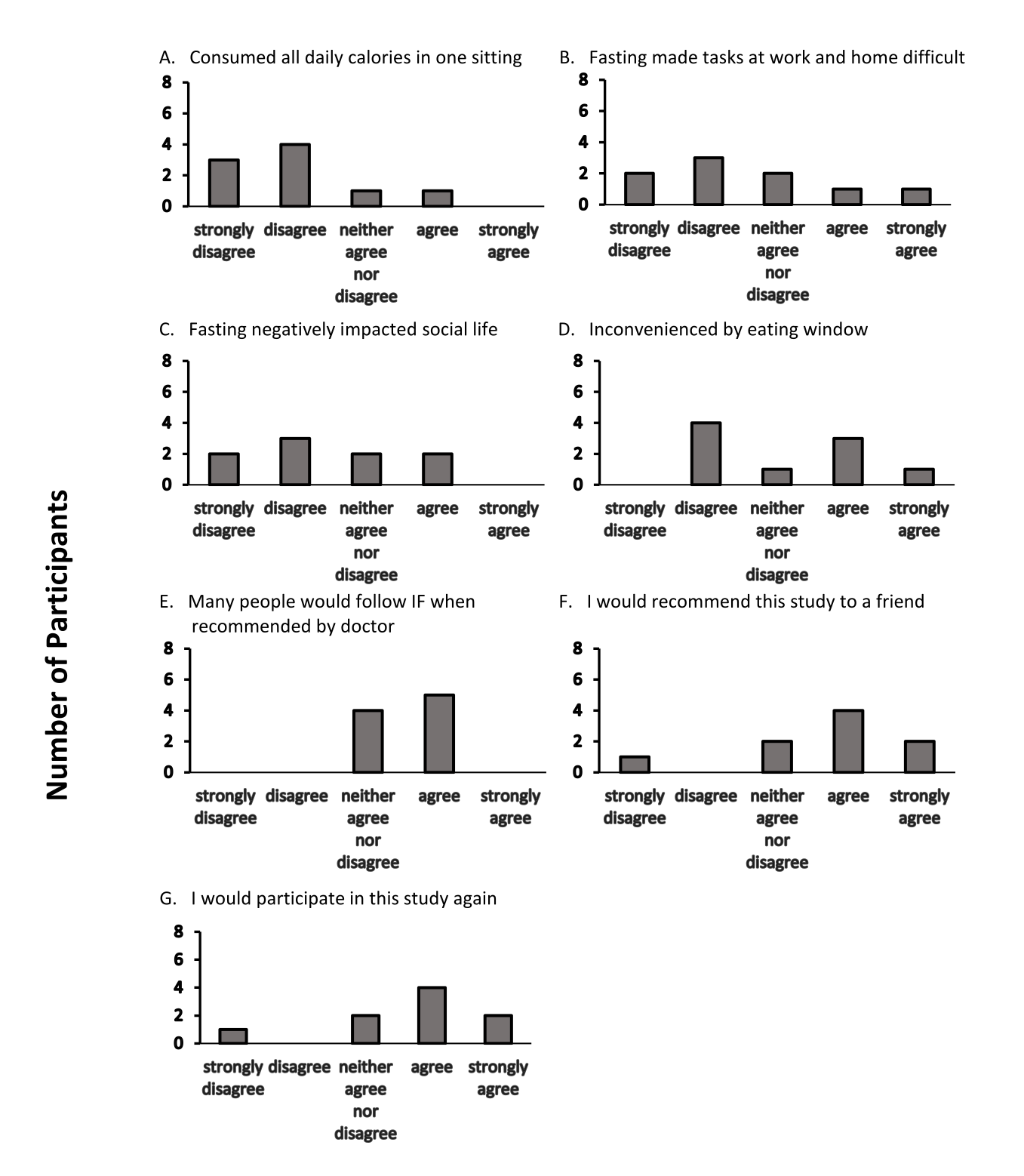

Supplement: Supplementary file 1 [file nutrients-12-00874-s001.zip › Supplementary Files/Figure S5 Participant responses to the seven questions within the socio-environmental domain of the Diet Satisfaction Survey.png]
